# Vitamin A depletion induced by cigarette smoke is associated with an increase in lung cancer-related markers in rats

**DOI:** 10.1186/s12929-015-0189-0

**Published:** 2015-10-14

**Authors:** Yuan Xue, Ethan Harris, Weiqun Wang, Richard C. Baybutt

**Affiliations:** Department of Human Nutrition, Kansas State University, 213 Justin Hall, Manhattan, KS 66506 USA; Department of Applied Health Science, Wheaton College, 501 College Avenue, Wheaton, IL 60187 USA

**Keywords:** Carcinogenesis, RAR α, RAR β, cJun, PCNA, Cyclin, Cigarette smoke, Retinoic acid

## Abstract

**Background:**

We have previously demonstrated that cigarette smoke is associated with a significant reduction of retinoic acid in rat lungs and the formation of tracheal precancerous lesions. However, the underlying mechanism of cancer risk induced by vitamin A deficiency is unclear. The purpose of this study was to determine whether the cigarette smoke-induced depletion of vitamin A is related to changes in lung cancer risk-related molecular markers.

**Results:**

We investigated the roles of the retinoic acid receptors (RARs) as well as other biomarkers for potential cancer risk in the lungs of rats exposed to cigarette smoke. Twenty-four male weanling rats were fed a purified diet and divided equally into four groups. Three experimental groups were exposed to increasing doses of cigarette smoke from 20, 40 or 60 commercial cigarettes/day for 5 days/week. After 6 weeks, the retinoic acid concentrations in the lung tissue as measured via high performance liquid chromatography (HPLC) significantly decreased (*P* < 0.01) in cigarette smoke exposed groups. Western Blot analysis revealed that cigarette smoke exposure increased lung protein expression of RAR α in a threshold manner and decreased RAR β and RAR γ expression in a dose-dependent fashion. Protein expressions of cyclin E and proliferating cell nuclear antigen (PCNA) were increased significantly in a dose-dependent manner in cigarette smoke exposed-groups. Additionally, there was a significant increase in protein expression of cJun and cyclin D1 demonstrating a threshold effect similar to that exhibited by RARα, suggesting a potential independent signaling pathway for RARα in lung carcinogenesis.

**Conclusions:**

Findings from this study suggest that cigarette smoke-induced lung retinoic acid depletion may involve two independent pathways, RARα- and RARβ-mediated, responsible for the increased cancer risk associated with cigarette smoke-induced vitamin A deficiency.

## Background

Lung cancer mainly caused by cigarette smoking remains the leading cause of cancer death in both men and women in the United States [1]. Understanding the mechanisms of cigarette smoke-induced lung cancer is an important area of research. Very little is known about how micronutrients such as vitamin A play a role in protecting against lung cancer. Two large clinical trials, the α-Tocopherol β-Carotene Cancer Prevention (ATBC) Trials and the β-Carotene and Retinol Efficiency Trial (CARET), found that smokers supplemented with β-carotene had an increased risk for developing cancer when compared to smokers without β-carotene supplementation [2]. A research group at Tufts University found that when ferrets, having β-carotene metabolism similar to humans, were supplemented with β-carotene and exposed to cigarette smoke, lung concentration of retinoic acid was reduced as was RAR-β expression, but cell proliferation markers were elevated [2]. The investigators suggested that the adverse effects upon vitamin A status were largely attributed to the interaction of the high dose of β-carotene and the cigarette smoke. To our knowledge no one has determined the effect of increased doses of cigarette smoke on lung retinoic acid and lung cancer risk-related molecular markers using a rat model. Further research in this area should impove our understanding of the underlying mechanisms for cigarette smoke-induced cancer and may lead to new strategies for decreasing lung cancer risk.

Vitamin A deficiency has been reported to cause preneoplastic changes in tracheal [3] and bronchial epithelium and to be associated with an increased risk of lung cancer [4, 5]. The loss of retinoic acid receptor beta (RARβ), one of the vitamin A nuclear receptors, is partly responsible for the lung carcinogenesis in vitamin A deficient individuals [6]. Retinoic acid receptor β is believed to promote transcription of tumor suppressor genes and has been found to be decreased in many preneoplastic lung cancers [7, 8]; therefore, decreased expression of RARβ has been suggested as a biomarker for lung cancer risk. How cigarette smoke-induced depletion of lung retinoic acid alters RARβ in rats is not known.

In contrast to the protective effects of RARβ, studies have linked increased expression of RARα with formation of tumors of the breast [9] and cervix [10], potentially by way of an estrogen-mediated pathway [9]. Additionally, vitamin A deficiency is linked with an *increased* expression of RARα, in contrast with the decreased expression of RARβ [11]. The precise mechanism of how RARα over-expression increases risk of lung cancer-related tumor formation is presently unknown.

Our previous study found that exposure to cigarette smoke decreased vitamin A level in lung tissue and was associated with precancerous lesions in the trachea [3]. To further understand the underlying mechanisms by which vitamin A deficiency might induce lung cancer, eight proteins including retinoic acid receptors, cell cyclins, proliferation markers, and nuclear transcription factors were selected and their nuclear expression was measured by Western blot.

Specifically, we investigated how the vitamin A depletion induced by cigarette smoke is related to decreased expression of RARβ, a potential cancer risk factor. Since dysregulation of the cell cycle is a prerequisite for the formation of most malignant tumors [12], we further investigated the impact of exposure to cigarette smoke on cell cycle checkpoints. Cyclins are one of the checkpoints in cell cycle, and their abundance is rate-limiting for progression through the different stages of the cell cycle. Another molecular marker for cell proliferation and cancer is nuclear transcription factor activator protein-1 (AP-1). AP-1 is a dimeric transcription factor that consists of homodimers and heterodimers of Jun (c-Jun, JunB, and JunD) and Fos (c-Fos, FosB, Fra1, and Fra2) [13, 14] or other transcription factors and proteins. The c-Jun has been found to play an integral role in lung cancer formation [15], yet its precise role in the signaling mechanism and its relation to the RAR pathway(s) remains unclear. Thus, the objective of this study was to determine how cigarette smoke-induced lung retinoic acid depletion altered lung RARs and their relationship to cancer-related cell proliferation markers and cyclin expression. We exposed rats to increasing doses of cigarette smoke for six weeks and then quantified lung retinoic acid and the protein expression of RARs, cell proliferation markers, and cyclins.

## Methods

### Animals and treatment

Male Sprague–Dawley weanling rats (range of weights: 50–75 g, Harlan Sprague Dawley, Indianapolis, IN) were housed individually in stainless steel cages at room temperature, 24 °C, under a 12-h light:dark cycle (light 0600–1800 h) with a relative humidity of 50 %. Male rats were chosen because they were previously shown to develop precancerous lesions in response to cigarette smoke-induced vitamin A deficiency. It will be important to evaluate these effects in female rats in future experiments. Animal care and use were approved by the Institutional Animal Care and Use Committee of Kansas State University. Rats were fed a standard AIN-93G diet [16] and water. Food intake was recorded daily and body weight was measured weekly. All groups were pair-fed throughout the entirety of the experiment. The cigarette smoke-treated rats were pair-fed to match the intake of the control group, which was the group that was exposed to air. This was to control for any potential reduced food intake of the cigarette smoke-exposed rats.

### Cigarette smoke exposure

Approximately one month old rats were randomly divided into four groups of six rats per group, and were exposed to air (control), one-pack, two-packs, and three-packs of cigarettes daily (nonfiltered commercial cigarette, 20 cigarettes/pack) for six weeks. The cigarette smoke treatment was modeled after previously published studies [3]. To model cigarette smoke exposure, rats were placed in a plastic chamber that was 65 cm long, 50 cm wide, and 45 cm high with three holes, two for holding two cigarettes at one end of the chamber and another hole on the opposite side of the chamber connected to a tube attached to a Leeson vacuum pump (model # A6C17EB20.1; Labconco, Kansas City, MO). The rats were exposed to the cigarette smoke of four standard cigarettes for 5 min followed by 5 min of air until all the cigarettes assigned to the group were consumed. One pack of cigarettes was considered one session and at least two hours of break were given between sessions. The extent of cigarette smoke exposure was ascertained by measuring total particulate matter inhaled and is noted in our previous study [3].

### Measurement of level of retinoic acid in lung by high performance liquid chromatography (HPLC)

Lungs were collected under yellow lights following termination of the study and immediately frozen in liquid nitrogen and kept at −70 °C until analysis. The lipid components were extracted from minced tissue, ~200 mg by a standardized method developed by Barua and Olson [17]. Tissue was ground in liquid nitrogen and then transferred to 10 mL of 2-propanol-dichloromethane (2:1, v/v) plus 0.1 % butylated hydroxytoluene (1 mg/mL). Twenty μL of retinyl acetate was added as the internal standard to the solution. The mixture was vortexed for 1 min and then stored under argon at −20 °C overnight. The following day, the mixture was vortexed again and returned to freezer. On the third day, the mixture was vortexed, and then filtered through 0.45 um syringe filter. The filtrate was evaporated to dryness under nitrogen. The residue was dissolved in 200 μL 2-propanol-dichloromethane (2:1, v/v). An aliquot of 50 μL was injected onto the HPLC system for analysis according to the procedure by Barua and Olson [17]. The concentration of retinoic acid was determined using the Beckman System Gold software (Beckman Instruments, Fullerton CA) and a 3-*μ*m Microsorb-MV column (4.6 x 100 mm) (Rainin, Woburn, MA), which was preceded by a guard column of C_18_. The solvent system consisted of methanol–water (3:1, v/v) containing 10 mM ammonium acetate (solvent A) and methanol-dichloromethane (4:1, v/v) (solvent B). The flow rate was 0.8 mL/min. A linear gradient was formed from solvent A to solvent B over a period of 15 min, followed by isocratic elution with solvent B (100 %), for another 15 min. At the end of the run, the gradient was reversed to initial conditions by applying a linear gradient of 5 min. The column was then allowed to equilibrate for 10 min with solvent A before the next run. Detection was monitored at 340 nm (Model 166, Beckman Instruments, San Ramon, CA). Under these conditions, all-trans retinoic acid and, all-trans retinyl acetate and all-trans retinyl palmitate was eluted at ~10.5, 12.1 and 15.7 mins, respectively. The recovery rates were 91 % for all-trans retinoic acid, 94 % for all-trans retinyl acetate and 92 % for all-trans retinyl palmitate

### Nuclear extraction procedure

The right lobe of the lung was immediately frozen at the end of the study and the tissue stored at 70 °C until analysis. In previous studies we used the right lobe of the lung for biochemical analysis and the left lobe for pathological evaluations [3]. The lung tissue nuclei were extracted as follows. Approximately, 0.15 g (1/3 of the right lobe) was placed in ice cold buffer A (20 mM Tris/HCl pH 7.8, 1 mM EDTA, 0.1 M NaCl, 1 mM phenylmethylsufonyl fluoride, and 1 μg/mL of each of the following: aprotinin, leupeptin and pepstain) and homogenized with a glass-teflon homogenizer (Con-Torque Eberbach Company Ann Arbor, MI). The homogenate was then centrifuged at 2000 × g for 15 min (Allegra 25R, Beckman Coulter). The supernatant was then removed, and the pellet was re-extracted using buffer B (buffer A with NaCl increased to 0.6 M) by tearing the pellet and vortexing in order to maximize the nuclear amount in the sample. The solution was then covered with mineral oil and spun at 190,000 × g using an ultracentrifuge (Optima L-90 K, Bechman Coulter). The supernatant was then removed by syringe and stored at −70 °C until analysis. Protein concentration was quantified using a spectrophotometer (UV-1601PC, Shimadzu).

### Western blot

Approximately 50 μg of nuclear extract in Tris lysis buffer was separated on 1.5 mm 12 % formaldehyde minigels. Protein concentration was measured by the Bio-Rad protein assay (Bio-Rad, Hercules, CA). The sample volumes were calculated to produce equal protein content. The protein was then transferred to a nitrocellulose membrane (Bio Rad, Hercules, CA) using Tris-Glysine Buffer pH 8.3 and blocked one hour at room temperature in a 5 % dry milk PBS solution. Antibodies against retinoic acid receptors α, β, γ, cyclin D1 and E, proliferating cell nuclear antigen (PCNA), and c-Jun and c-Fos were purchased from Santa Cruz Biotechnology (Santa Cruz, CA). An antibody against β actin was used for loading control (data are not provided in this paper). Antibodies after dilution recommended by the manufacturer were incubated overnight at 4 °C. The blots were washed and then incubated with secondary antibody (1:1000, Santa Cruz, CA). The blots were visualized using Chemi Glow West (Alpha Innotech Corporation) on a Fluorochem 8800 (Alpha Innotech Corporation) imaging system. The degree of expression of immunodetected signaling molecules was measured by densitometry.

### Statistical analysis

All data are presented as means ± SEM. Treatment effects were analyzed using one-way ANOVA with the general linear model procedure (SAS Institute, Cary, NC). In all analyses, *P* < 0.05 was considered significant. The correlation coefficients of each pair of groups are calculated in SAS PROC CORR.

## Results

### Concentrations of retinoic acid were decreased in lungs exposed to cigarette smoke

**Fig. 1:**
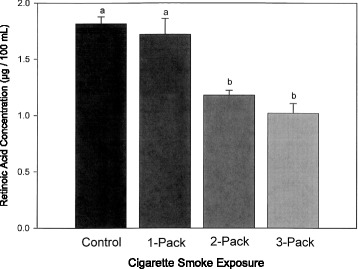
Retinoic Acid Level in Lungs Exposed to Cigarette Smoke. The all-trans retinoic acid level in lungs exposed to different doses of cigarette smoke for 6 weeks. Values are mean ± SEM, *n* = 6. Groups with different letters are significantly different, *P* < 0.05

All animals completed the cigarette smoke exposure treatment. The concentration of retinoic acid in lungs was decreased when exposed to 2-pack and 3-pack of cigarettes smoke (*P* < 0.021, Fig. 1), but not significantly different for the treatment group exposed to one pack of cigarettes when compared to the control group.

### Expressions of RARβ and RAR γ were decreased in lungs exposed to cigarette smoke

**Fig. 2:**
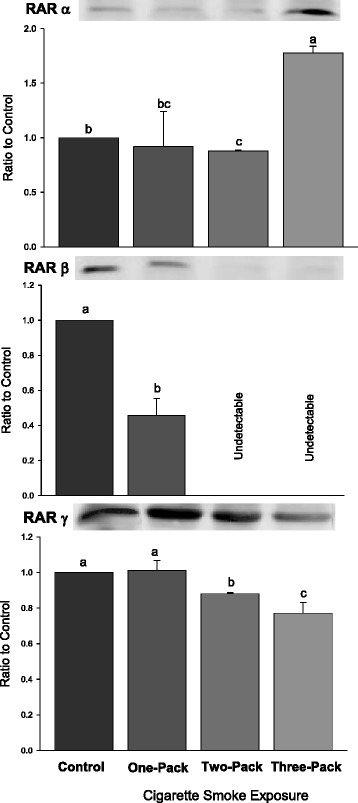
Western Blot Analysis for RAR Levels. Protein expressions of retinoic acid receptors (RARs: RARα, β, γ) in lungs exposed to different doses of cigarette smoke for 6 weeks. Representative Western Blot analyses are shown that used anti-RARα, anti-RARβ and anti-RARγ antibodies. The size of the detected RARα, RARβ and RARγ were all 53 kilodaltons (kDa). The intensity of the protein signal was determined by densitometry analysis (three samples in each group). The relative protein values in the three treatment groups were calculated as a mean value ± standard deviation (SD) of the data relative (ratio) to control with the control set at 1.0. Different letters represent significant difference among groups (P < 0.05)

The expression of RARβ was significantly reduced by more than 50 % in the group that was exposed to one pack of cigarettes and undetectable in the other two cigarette smoke-treated groups using Western blot analysis. A representative sample of RARβ protein expression is depicted in Fig. 2. High doses of cigarette smoking exposure (2 and 3 packs) significantly (*P* < 0.05) decreased RAR γ expression.

### Expression of RARα was increased in lungs exposed to a three pack dose of cigarette smoke

The expression of RARα was significantly increased in the group that was exposed to three packs of cigarettes using Western blot analysis (*P* < 0.038). A representative sample of RARα protein expression is depicted in Fig. 2. There appears to be a dose threshold for the RARα lung response when exposed to increasing amounts of cigarette smoke.

### Expressions of proliferation indicators: PCNA and cJun were increased

A strong proliferative response in lungs either indicated by PCNA or by cJun was observed in cigarette smoke-treated groups. The expression of cJun significantly increased only in the treatment group that was exposed to 3-pack of cigarette smoke (Fig. 2, *P* < 0.001). There was a dose-dependent response found between cigarette smoke exposure and PNCA. The expression of PCNA was increased when the dose of cigarette smoke exposure was increased (*P* < 0.001).

### Cyclins E and D1 were increased in lungs exposed to cigarette smoke

**Fig. 3:**
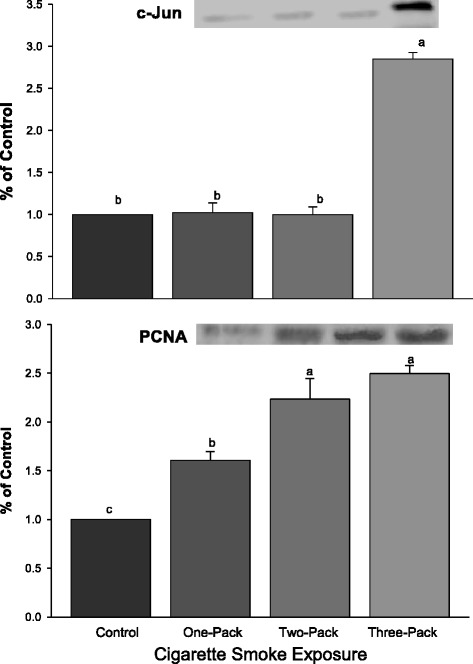
Western Blot Analysis for cJun and PCNA. Protein expressions of cJun and PCNA in lungs exposed to different doses of cigarette smoke for 6 weeks. Representative Western Blot analyses are shown that used anti-cJun, and anti-PCNA antibodies. The size of the detected cJun and PCNA were 39 kDa and 36 kDa, respectively. The intensity of the protein signal was determined by densitometry analysis (three samples in each group). The relative protein values in the three treatment groups were calculated as a mean value ± standard deviation (SD) of the data relative (ratio) to control with the control set at 1.0. Different letters represent significant difference among groups (P < 0.05)

Two G1 phase cyclins were investigated in this study. Cyclin E was found to be significantly increased in all three treatment groups (Fig. 3, *P* < 0.05), exhibiting a dose-dependent response. However, for cyclin D1, there was no dose-dependent response, and only the group exposed to the highest dose of cigarette smoke had significantly increased expression compared to the control group and other two groups.

### Linear correlation coefficients between levels of gene expression among different groups

**Fig. 4:**
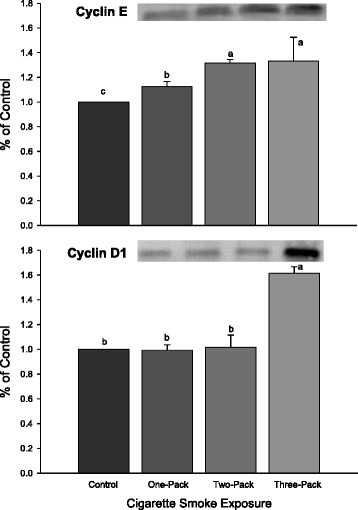
Western Blot Analysis for Cell Cyclins. Protein expressions of cyclins E and D1 in lungs exposed to different doses of cigarette smoke for 6 weeks. Representative Western Blot analyses are shown that used anti-cyclin E, and anti-cyclin D1 antibodies. The size of the detected cyclin E and D1 were 50 kDa and 36 kDa, respectively. The intensity of the protein signal was determined by densitometry analysis (three samples in each group). The relative protein values in the three treatment groups were calculated as a mean value ± standard deviation (SD) of the data relative (ratio) to control with the control set at 1.0. Different letters represent significant difference among groups (P < 0.05)

The protein level of RARβ in rat lung was significantly decreased and directly correlated with the concentration of retinoic acid (*P* < 0.001) in response to increasing exposure to cigarette smoke. The responses of RARβ and retinoic acid to the increased cigarette smoke exposure were inversely related to the level of cyclin E (*P* < 0.001) and PCNA (*P* < 0.05), but not significantly associated with the expression of cyclin D1 or cJun (Table 1). Cyclin D1 was significantly correlated with cJun (*P* < 0.001) and with PCNA (*P* < 0.05). The cigarette smoke response curve for RARα was similar to that of cyclin D1 and cJun (Figs. 2, 3, and 4). There was no significant increase in RARα, cyclin D1, or cJun at the lower dose of cigarette smoke (one or two packs/d), but a significant increase in all three at the highest dose of cigarette smoke (three packs).

**Table 1:** Correlation Coefficients

Correlation for Retinoic Acid and Lung Cancer-Related Markers						
Measurements	RARβ	cJun	PCNA	cyclinD1	cyclinE	RA
RARβ	1.00	-	-	-	-	-
cJun	−0.53	1.00	-	-	-	-
PCNA	−0.89*	0.81*	1.00	-	-	-
cyclinD1	−0.50	0.98**	0.80*	1.00	-	-
cyclinE	−0.98**	0.56	0.90*	0.54	1.00	-
RA	0.96**	−0.53	−0.84*	−0.50	−0.95**	1.00

Linear correlation coefficients between levels of protein expression in the different groups. This table shows the linear correlations between levels of protein expressions and retinoic acid concentration among different groups. Correlation coefficients are designated by a star * and two star ** to indicate significance of *P* < 0.05 and *P* < 0.001, respectively. All other correlations were not significant

*RA* retinoic acid, *RAR* retinoic acid receptor, *PCNA* proliferating cellular nuclear antigen

## Discussion

In this study, we investigated how increasing exposure to cigarette smoke depleted retinoic acid in rat lung tissue and how this depletion was associated with increased lung cancer risk via changes in protein levels of specific biomarkers for cell proliferation and cell cycle transition. It is well-known that cigarette smoke contains numerous radicals that may deplete vitamin A, including both carbon-centered and oxygen-centered products from NO/NO_2_ reactions with reactive compounds such as isoprene in smoke. In addition, benzopyrene, a carcinogen in cigarette smoke, has been found to deplete lung and liver vitamin A levels when rats are fed benzopyrene [18]. We found that cigarette smoke exposure decreased both the lung retinoic acid concentration and the protein levels of RARβ in a dose-dependent manner while increasing expression of RARα in a threshold-dependent response. Expression of two cell proliferation markers, cJun and PCNA, increased. In addition, the expression of two G_1_ phase cyclins, D1 and E, were increased in response to cigarette smoke exposure.

This study presents the first evidence that six-week exposure to one, two, and three packs of cigarettes per day, five days per week, depletes lung retinoic acid in rats and is associated with increasing molecular markers for cancer while depleting the tumor suppressor RARβ. In a previous study, we exposed rats to two packs of cigarettes per day for four weeks and used microarray analysis to discover that dietary retinoic acid down-regulated genes that promoted cell divisions/proliferation in response to cigarette smoke [19]. Additionally, we have shown that two-pack cigarette exposure per day over six weeks depleted lung retinol and was associated with precancerous tracheal lesions and emphysema [3]. Cigarette smoke-induced depletion of lung retinoic acid has been reported in ferrets [8]. In addition, airborne particulate matter which contains pentachlorophenol, also in cigarette smoke, depleted lung vitamin A [20] as did benzopyrene the cigarette smoke carcinogen [18].

### Role of RARβ in lung cancer

RARβ expression was decreased in one-pack of cigarette smoke exposure and undetectable in two-pack and three-pack doses. Because many studies suggest a tumor suppressor role for RARβ [21] and reduced RARβ expression is found in about 45-50 % of lung cancers [22, 23], the decreased expression of RARβ observed in this study suggests a greater risk for development and/or promotion of lung cancer. The reduction in RARβ expression may be caused by genomic loss or epigenetic silencing due to genomic methylation or histone deacetylation [24–26]. Hypermethylation of the RARβ promoter region is an early event in lung carcinogenesis and is one of the most frequent methylation defects detected in the histopathologically normal bronchial epithelium of heavy smokers [27]. Thus, the loss of RARβ expression may be an early event leading to lung carcinogenesis [22, 28].

The concentration of retinoic acid was decreased significantly in the cigarette smoke-exposed groups which strongly correlated with the decreased expression of RARβ. The decreased retinoic acid likely preceded the loss of RARβ expression since others have found that all-trans-retinoic acid is a potent inducer for mRNA for RARβ [29]. We previously found that the depletion of vitamin A is associated with precancerous lesions in cigarette smoke-exposed trachea [3]. It has long been known that retinoic acid has a cancer-preventative effect because vitamin A-deficient rodents develop squamous metaplasia, a precancerous lesion [30]. More recently, it has been discovered that lung RARβ levels return to normal if rats that were previously fed a vitamin A-deficient diet were supplemented with retinoic acid [11]. As previously mentioned, we have found that dietary retinoic acid can reverse some of the increased lung cancer-related gene expression in response to cigarette smoke in rats [19]. The role of vitamin A in protection against cigarette smoke-induced cancer is an important area for further research.

Because RARβ acts as a tumor suppressor gene, its reduced expression could lead to enhanced cell proliferation and potentially to tumor formation. It has previously been shown that an antagonist to RARβ can abolish the retinoic acid inhibition of mitogenic pathways, extracellular signal regulated kinase (ERK 1/2), and cJun N-terminal kinase (JNK) in human scleral fibroblasts [31]. In the present study, a dose dependent increase in the level of a marker of cell proliferation, proliferating cell nuclear antigen (PCNA), in cigarette smoke-exposed groups was observed. Expression of one of the AP-1 proteins, cJun, also was increased and strongly correlated with the expression of PCNA. Overexpression of AP-1 is found to be associated with squamous metaplasia and with increased expression of PCNA in the lung [8]. Mechanistically, the RARβ DNA binding domain was important for inhibition of AP-1 activity [32]. In addition, it is found that RARβ has unique anti-AP-1 activity in that it exhibits strong inhibition of AP-1 activity via protein-protein interaction in the absence of the ligand retinoic acid [33, 34]. It is interesting to note that in the present study the increased RARα response closely resembles the increased cJun response to the smoke exposure, whereas there did not appear to be a significant inverse relationship between RARβ and cJun suggesting independent responses for RARα and RARβ to the cigarette smoke.

### Role of RARα in Lung Cancer

Decreases in retinoic acid increase AP-1 transcriptional activity [34] and thereby increases the expression of cJun genes. Retinoic acid is also found to down-regulate the transcriptional activity by cJun in bacteria [35]. Findings of the present study suggest that an increase cJun-mediated AP-1 transcriptional activity observed in retinoic acid-deficient, cigarette smoke-exposed rat lungs can be more attributed to an agonistic mechanism via RARα over-expression than reduction in RARβ-mediated antagonistic response. cJun levels measured in rat lungs closely replicated the threshold-response demonstrated by RARα expression in response to the cigarette smoke exposure. No significant increases were observed until the dose of cigarette smoke exposure reached 3 packs/d. This apparent parallel response suggests a cooperative interaction of RARα and AP-1 as reported previously using T47D breast cancer cells [36].

### Role of cell cyclin irregularity in lung cancer

Next, we investigated cyclins involved in promoting G_1_ phase to S phase transition to observe changes in factors related to cell cycle regulation. In the majority of cells/tissues that have been examined, cell cycle regulation occurs in the G_1_ phase. Moreover, the connection between cyclins and cancer has been substantiated with G_1_-type cyclins since G_1_ phase to S phase progression is a key factor in carcinogenesis [12].

#### Cyclin E

Cylin E regulates late G1 and early S phases [37]. Cyclin E, forming complexes with cyclin-dependent kinase 2, is essential for G1/S transition and has a profound role in oncogenesis. Both cyclin E and D1 were overexpressed in the lung exposed to heavy cigarette smoke in the present experiment. The overexpression of cyclin E and D1 in the lungs of rats exposed to cigarette smoke suggests a promotion of S phase entry for cell proliferation.

In this study, only the decrease of RARβ and increase of cyclin E and PCNA were dose-dependent with regard to cigarette smoke exposure, suggesting a potential relationship. These results indicate that cigarette smoke exposure plays a significant role in these alterations. Cyclin E levels determine the time point when cells enter S phase [37, 38]. In addition, of the two cyclins measured in this study, only cyclin E was strongly associated with the concentration of retinoic acid and level of RARβ. Together, these data suggest that increased cell proliferation in response to increased expression of cyclin E is associated with the decrease of RARβ in lungs exposed to cigarette smoke.

#### Cyclin D1

Unlike cyclin E, cyclin D-type cyclins are synthesized in early G_1_ phase. Previous studies have reported an increase in cyclin D1 expression in non-small-cell lung cancer (NSCLC) cases [39]. cJun is reported to promote G_1_ phase progression through direct transcriptional control of cyclin D1 [40].

Our study suggested that, similar to RARα, cyclin D1 expression increased in a dose-dependent manner according to increasing cigarette smoke exposure; no significant increases were demonstrated in cyclin D1 levels until cigarette smoke exposure reached 3 packs/d. Cyclin D1 levels measured across the treatment groups were inversely related to lung retinoic acid levels (*P* < 0.05). Cyclin D1 levels were also very strongly associated with cJun levels measured by Western Blot (*r* = 0.98, *P* < 0.001). Together, these findings suggest that RARα, cJun, and cyclin D1 act in concert to promote cigarette smoke induced cell proliferation independently of RARβ.

### Role of RARγ in lung cancer

RARγ levels in the lung were shown to decrease in a dose-dependent fashion with increasing cigarette exposure (Fig. 2). RARγ has been associated with elastin production [41], and its reduced expression appears to be associated with the development of emphysema [42]. Decreased RARγ has also been found to be depleted in 41 % of non-small-cell lung cancer [43]. The precise role for RARγ in cigarette smoke-induced lung cancer needs further investigation.

## Conclusion

Cigarette smoke-induced retinoic acid deficiency was found to be highly associated with multiple molecular biomarkers of increased lung cancer risk in this study. Increasing cigarette smoke exposure depleted lung retinoic acid and decreased RARβ and RARγ expression in a dose-dependent fashion. The decreased RARβ expression was associated with a dose-dependent increase in cyclin E and PCNA. The smoke exposure increased RARα in a curvilinear fashion. This curvilinear response was also observed for cyclin D1 and cJun. Two independent signal cascades mediated by decreased RARβ and increased RARα expression may contribute to the precancerous lesions observed from our previous study [3]. Whether the transcription regulation in this model through epigenetics or transcription factor:DNA interactions as measured by a ChIP assay and whether the addition of dietary vitamin A in conjunction with cigarette smoke exposure prevents and/or decreases lung cancer risk are worth future exploration.

## Abbreviations

CKI: Cyclin Kinase Inhibitor
HPLC: High Pressure Liquid Chromatography
NSLC: Non-Small Cell Lung Cancer
PCNA: Proliferating Cell Nuclear Antigen
RAR: Retinoic Acid Receptor
